# Responsible Research and Innovation Framework, the Nagoya Protocol and Other European Blue Biotechnology Strategies and Regulations: Gaps Analysis and Recommendations for Increased Knowledge in the Marine Biotechnology Community

**DOI:** 10.3390/md20050290

**Published:** 2022-04-26

**Authors:** Xenia Theodotou Schneider, Belma Kalamujić Stroil, Christiana Tourapi, Céline Rebours, Susana P. Gaudêncio, Lucie Novoveska, Marlen I. Vasquez

**Affiliations:** 1 XPRO Consulting Limited, Strovolos 2021, Cyprus; 2 Institute for Genetic Engineering and Biotechnology, University of Sarajevo, 71000 Sarajevo, Bosnia and Herzegovina; belma.kalamujic@ingeb.unsa.ba; 3 Department of Chemical Engineering, Cyprus University of Technology, Limassol 3036, Cyprus; ctourapi@yahoo.com (C.T.); marlen.vasquez@cut.ac.cy (M.I.V.); 4 Møreforsking AS, 6021 Ålesund, Norway; celine.rebours@moreforsking.no; 5 Associate Laboratory i4HB, Institute for Health and Bioeconomy, NOVA School of Science and Technology, NOVA University Lisbon, 2819-516 Caparica, Portugal; s.gaudencio@fct.unl.pt; 6 UCIBIO—Applied Molecular Biosciences Unit, NOVA School of Science and Technology, NOVA University of Lisbon, 2819-516 Caparica, Portugal; 7 Ocean4Biotech, Edinburgh EH12 5AD, UK; lucie.novoveska@gmail.com; 8 European University of Technology, Limassol 3036, Cyprus

**Keywords:** marine biotechnology, blue biotechnology, sustainable blue economy, convention on biological diversity, Nagoya Protocol, Responsible Research and Innovation (RRI), policy recommendations, legal and ethical compliance workflow, marine natural products, marine micro- and macro-organisms, marine genetic resources

## Abstract

As the quest for marine-derived compounds with pharmacological and biotechnological potential upsurges, the importance of following regulations and applying Responsible Research and Innovation (RRI) also increases. This article aims at: (1) presenting an overview of regulations and policies at the international and EU level, while demonstrating a variability in their implementation; (2) highlighting the importance of RRI in biodiscovery; and (3) identifying gaps and providing recommendations on how to improve the market acceptability and compliance of novel Blue Biotechnology compounds. This article is the result of the work of the Working Group 4 “Legal aspects, IPR and Ethics” of the COST Action CA18238 Ocean4Biotech, a network of more than 130 Marine Biotechnology scientists and practitioners from 37 countries. Three qualitative surveys (“Understanding of the Responsible Research and Innovation concept”, “Application of the Nagoya Protocol in Your Research”, and “Brief Survey about the experiences regarding the Nagoya Protocol”) indicate awareness and application gaps of RRI, the Nagoya Protocol, and the current status of EU policies relating to Blue Biotechnology. The article categorises the identified gaps into five main categories (awareness, understanding, education, implementation, and enforcement of the Nagoya Protocol) and provides recommendations for mitigating them at the European, national, and organisational level.

## 1. Introduction: Contextual Perspective for the Identification of Gaps and Provision of Recommendations to the Marine Biotechnology Community

The European Union (EU) refers to Marine Biotechnology (MB) as Blue Biotechnology (BB) and considers BB as an essential innovation sector for the EU Blue Growth Strategy [1]. Blue Biotechnology is a multidisciplinary, knowledge- and capital-intensive technological area that significantly contributes to innovations in the pharmaceutical, medical, cosmeceutical, nutraceutical, aquacultural, agricultural, and energy sectors [2]. The Blue Bioeconomy and Blue Biotechnology sectors in Europe include the non-traditionally commercially exploited marine organisms and their biomass applications. Macro- and micro-algae, bacteria, fungi, invertebrates, and vertebrates are among the important marine resources used as feedstock in the Blue Bioeconomy [3]. The global BB market is estimated to reach EUR 6.64 billion by 2026, growing at a Compound Annual Growth Rate (CAGR) of 8.7% during 2021–2026 [4]. In 2020, Europe had the largest share (37%) of the global BB market due to growing research and innovation (R&I) activities [3]. For example, the algae sector in Europe currently employs approximately 14,000 people and has an economic value of EUR 1.69 billion [3]. In recognition of the potential of BB, the European Parliament has created public/private partnership funds for investments into various initiatives, i.e., EUR 3.7 billion for the Bio-Based Industries Joint Undertaking (BBI-JU). BBI-JU was also endorsed in 2021 with an additional EUR 2 billion to create more than 13,000 jobs in the three priorities that encompass Circular Blue Bioeconomy, which includes BB. Furthermore, the European Commission (EC) has approved EUR 234 million for BB under the R&I funding programme Horizon Europe for 2021–2027 [3].

With the rise of new opportunities in the EU and global BB sector, the needs and requirements for Responsible Research and Innovation (RRI) are exponentially growing to ensure societal relevance and ethical, responsible, and sustainable financial returns. RRI aims at enabling research and innovation results to meet societal needs and challenges through sustainability. Furthermore, innovation investments aim at protecting the intellectual property rights (IPR) and patent disclosure requirements (PDRs) for improved capitalisation. At the same time, investors are evaluating the sustainability of potential innovations based on their environmental sustainability and protection of ocean health and conservation of biodiversity. Consequently, applying the EU and international regulatory frameworks is necessary for ensuring responsible and sustainable innovation without jeopardising marine biodiversity and adding extra human pressures to the already threatened marine environments.

The most relevant and crucial global policy framework addressing the above is the Convention on Biological Diversity (CBD, 1992) with its derivatives: Access and Benefit Sharing (ABS, 2014) and the Nagoya Protocol (NP, 2014) on “Access to Genetic Resources and Fair and Equitable Sharing of Benefits Arising from their Utilisation”. The CBD defines the access and benefit-sharing provisions, which are designed to ensure that the physical access to genetic resources is facilitated and that when benefits are obtained from their use, these are shared equitably with the genetic resource providers. The EU complied with the NP through the Regulation *EU No 511/2014*. However, researchers perceive such legislation as a hurdle instead of an enabler. Researchers also lack the knowledge that many countries require compliance with regulations for achieving intellectual property protection as, for example, in a patent, and that poor or non-compliance may jeopardise their R&I results. Several scientists are unaware that to achieve a patent, information will be openly disclosed and thus exposed to misconduct, misappropriation, or misuse of Genetic Resources (GR) and Traditional Knowledge (TK). Realising possible non-compliance with legislation, countries require applicants during patent application examination to disclose the origin and compliance of GR and TK [5].

Considering all the above, the Working Group 4 (WG4) “Legal aspects, IPR and Ethics” of the Ocean4Biotech COST Action CA18238 [6] reviewed the status of awareness and knowledge relating to BB’s application of RRI, IPR, NP, and the EU-related policy frameworks and regulations. The WG4’s goals are to: (1) increase awareness of the most important regulations and policies that relate to BB R&I; (2) present a value-chain overview and easy access to regulations and policies at the EU level; (3) demonstrate the variability in the NP implementation in Europe; (4) draw attention towards RRI and embedding it in R&I processes; (5) highlight gaps; and (6) provide recommendations for improving awareness and knowledge.

This perspective paper is based on a revision of regulatory frameworks applied to the BB sector in Europe and the conclusions from three qualitative surveys conducted by WG4 in the Ocean4Biotech—a European transdisciplinary networking platform for the marine biotechnology network—to investigate: (1) awareness and knowledge towards the NP; (2) the extent of NP implementation in EU member states and associated countries; and (3) knowledge and application of RRI in R&I processes. Through these surveys, several gaps have been identified concerning NP awareness at the academic level, as well as in private and public research organisations, spin-offs, and SMEs. This article highlights the identified gaps and suggests mitigation actions to enable the responsible viability and sustainability of R&I towards societal benefits and environmental protection.

The paper is divided into six sections: (1) overview of the EU regulatory frameworks relating to BB, (2) overview of the NP, (3) overview of the RRI, (4) survey results and identified gaps, (5) mitigation actions and recommendations, and (6) conclusions.

## 2. Overview of the European Union Regulatory Framework Relating to Blue Biotechnology

The European Commission (EC) recognised the importance of the BB sector, which was already included in its Blue Growth Strategy in 2012 [7] as one of five innovative and high-potential sectors. The EC recently published the Sustainable Blue Economy Approach [7], which adheres to the environmental and climate objectives of the European Green Deal. The Sustainable Blue Economy Approach expects coherent actions in all Blue Economy sectors, including BB, without harming the environment. At the same time, the approach highlights the need for investments in R&I and for upgrading skills and capacity. It highlights the need for reversing the overexploitation of resources and the destruction of natural habitats. Lastly, the term “Blue Growth” has been replaced by “The Sustainable Blue Economy Approach”, a term which further accentuates the necessity for the marine sectors to be responsible and sustainable and to provide positive social and environmental impacts and innovation, along with economic benefits [3].

The EC is organised in several Directorate-Generals (DGs) and executive agencies that develop, implement, and manage EU policies, laws, and funding research programmes. For instance, the DG MARE deals with Maritime Affairs and Fisheries, the DG AGRI with Agricultural and Rural Development, the DG RTD with R&I, the DG SANTE with Health and Food Safety, and the DG ENV with the Environment. Three of the executive agencies relevant to BB are the “European Climate, Infrastructure and Environment Executive Agency”, the “European Health and Digital Executive Agency”, and the “European Research Council Executive Agency”. As the BB sector delivers compounds and innovation possibilities to other sectors (such as nutraceuticals, pharmaceuticals, agriculture, new materials, and bioenergy), the directives and regulations that govern the BB sector originate from different DGs. Another aspect to be considered is that BB is part of the Smart Specialisation Strategies of Europe, which is a policy instrument favouring the discovery of innovation potential at the regional level, which provides funding possibilities through the European Regional Development Fund [1]. Moreover, it is funded through a variety of research programmes, such as Horizon 2020, Horizon Europe, and Bio-Based Industries Joint Undertaking (BBI-JU). Under the Blue Growth Strategy and now the Sustainable Blue Economy, BB was highlighted as an emerging sector for Europe and a vital innovation area for its regions, in contrast with the USA and China, where BB is already considered a major sector [7]. The challenge, however, is how to achieve the sustainable use of ocean resources without destroying and endangering its fragile ecosystems and safeguarding ocean health for future generations while, at the same time, meeting the demands generated by the increasing human population and its economic development. The extraction of living resources may affect the marine environment, for example, creating pressures on wild species’ populations (e.g., extraction, mortality, and injury), physically disturbing the seabed, creating an imbalance in marine biotopes, and/or producing sources of litter. Likewise, the cultivation of living resources may introduce microbial pathogens, spread non-indigenous species, and/or introduce organic matter, including pollutants such as xenobiotics that can destabilise the local marine ecosystem. In this respect, European and international policymakers [8] face the dilemma of responsibly and sustainably achieving economic growth.

In the past decade, the interest and focus on the health of the oceans and the seas has increased. It has accelerated since the 2017 “Ocean Conference of the United Nations” focusing on the UN Sustainable Development Goal 14 (SDG 14) “Life below water” [9], which has also been embraced by the EU. In December 2019, the EU launched the European Green Deal [10], which is a coordinated set of policies and legislations with the overarching aim of making Europe climate neutral by 2050. At the core of the European Green Deal is the EU Biodiversity Strategy for 2030 [11], a comprehensive and long-term action plan for protecting nature and reversing the degradation of ecosystems. Specifically, the EU Biodiversity Strategy for 2030 aims at protecting wildlife and fighting illegal wildlife trade. It highlights the importance of sustainably managing marine areas and reversing biodiversity loss by further pushing the adoption of the Convention on Biological Diversity (CBD). It will also prepare the EU to take a leading role in the upcoming international negotiations on a new global framework to halt biodiversity loss. EU policies aim at economic development activities becoming more biodiversity-friendly, focusing further on the sustainable use of ecosystems, supporting the recovery of European biodiversity, and restoring damaged ecosystems through the “do no significant harm” (DNSH) of the European Green Deal implementation. The DNSH has already been embedded in the Horizon Europe research and innovation funding programme. Furthermore, with the launch of the Sustainable Blue Economy Approach, the EU has pledged: (1) to protect at least 30% of the EU sea area from reversing biodiversity loss and (2) to minimise negative impacts on marine habitats [12]. This approach further highlights the value of biodiversity in economic terms and opposes the common belief that marine protected areas (MPAs) are diminishing economic development. Several studies highlighted that for every Euro invested in MPAs, the benefits increase by giving a triple return on investment, confirming that MPAs can generate significant economic benefits to individual sectors and in the wider local economies [13]. The EU aims at protecting and monitoring 30% of its seas by 2030, thus, expanding its MPAs to 10% of its marine surface. These new MPAs will be strictly protected, particularly those with seagrass meadows. The EU member states are responsible for enforcing the MPAs from 2020, and their full enforcement will have to be demonstrated by 2023. The EU has also declared zero-tolerance towards illegal practices and non-sustainable harvesting of marine resources [12]. Hence, the EU expects its member states to fully implement the EU Common Fisheries Policy, the Marine Strategy Framework Directive, and the Birds and Habitats Directives with a special focus on sensitive species and seabed habitats. For implementing these directives and policies, all member states were to finalise and deliver their national maritime spatial plans by 2021. This further reinforces the necessity for BB researchers and stakeholders to be aware and knowledgeable of the relevant regulations and ethics.

The EU objective is to actively participate in the global ambition that the world ecosystems are restored, conserved, and adequately protected by 2050 [7]. The EU and the CBD Parties will push for committing to the “net-gain” principle: “to give back to nature more than humans take” [13]. Natural resources are limited and fragile, and the human-induced extinction of species should be avoided at all costs. Furthermore, the EC intends to create the European Biodiversity Framework to map obligations and commitments at a national level and specify the roadmap for its implementation, including measurement, monitoring, and review. This new governance framework will be based on “co-responsibility” and “co-ownership” and will support administrative, capacity-building, transparency, stakeholder dialogue, and participatory governance at all levels. The EC has decided to use Non-Governmental Organisations (NGOs) to act as the supervisory body, and additional strengthening of the judicial system will ensure full compliance with the EU legislative framework and assurance [13].

The EU Taxonomy [14], a classification system establishing a list of environmentally sustainable economic activities, entered into force on the 12 July 2020 and envisages the scaling up of sustainable investments and the implementation of the European Green Deal. The EU Taxonomy Regulation provides to companies, investors, and policymakers appropriate definitions for economic activities that are considered environmentally sustainable. It is an instrument for creating security and protecting private investors from greenwashing, helping companies to become more climate-friendly, mitigating market fragmentation, and enabling investments to shift where they are most needed. The EU has established the basis for the EU Taxonomy by setting out overarching conditions for economic activities that must be met to qualify as environmentally sustainable. The EU Taxonomy Regulation also applies to the marine environment by specifically “ensuring the sustainable use of marine ecosystem services or contributing to the good environmental status of marine waters, by protecting, preserving or restoring the marine environment and by preventing or reducing inputs in the marine environment”. It establishes six environmental objectives to which an economic activity must positively and substantially contribute. These objectives are: (1) Climate change mitigation; (2) Climate change adaptation; (3) Sustainable use and protection of water and marine resources; (4) Transition to a circular economy; (5) Pollution prevention and control; and (6) Protection and restoration of biodiversity and ecosystem.

### Helping the Marine Biotechnology Researchers, Innovators, and other Stakeholders Navigate through the Regulations

Identifying and listing the regulations and policies governing the BB sector at the EU level has not proven to be an easy task. Even though BB is part of the European Sustainable Blue Economy Approach, it is a field that lags behind compared with other Blue Economy sectors, such as maritime, fisheries, aquaculture, and coastal tourism. In the quest to identify the current policy framework that governs the first research and development stages of BB, it appears that the knowledge of the researchers falls behind in several policy areas (e.g., the Marine Strategy Framework Directive [15], Marine Protected Areas [16], the Directive of Genetically Modified Microorganisms [17], and the Directive on the Legal Protection of Biotechnological Innovations [18]). It has also become evident that a BB policy knowledge platform or hub is missing at the EU level that provides researchers, innovators, and other stakeholders an overview of the regulatory requirements, responsibilities, and societal expectations towards BB. Despite the fact that BB is part of the Smart Specialisation Platform Eye@RIS3 “Innovation Priorities in Europe”, when searching “Blue Biotechnology” in the platform, there are no hits. A very limited number of entries describe the kind of support they could provide concerning BB, and none of them had any information on providing support regarding any policy framework. It would be further beneficial for researchers and regions if the Smart Specialisation Platform is expanded to provide an overview and guidance by using the Policy domains for policy dissemination and awareness.

Several regional initiatives address the needs of the above regulations and legislations and provide directions for achieving the Sustainable Blue Economy, such as the Baltic Sea Parliamentary Conference [19] and the Scanbalt association [20]. In the first initiative, the focus on BB was minimal compared to other sectors, such as maritime, coastal tourism, and bioenergy. In the second one, the focus on BB seems to be less evident from 2016 onwards. In the Mediterranean countries, the trend is even slower, as identified during the EU-funded project MARINA [21], which focused on RRI in all Sustainable Blue Economy sectors, including BB.

In this respect, herein we provide an overview of the EU and international regulations that govern the BB value chain, from organism discovery and the first research and development phases of the BB until the compounds are transferred to another sector. Table S1, in the Supplementary Material, outlines this and is intended to raise researchers’ and stakeholders’ awareness. This table is by no means exhaustive but provides the main regulations at the EU and international levels for the BB initial value-chain stages.

## 3. Overview of the Nagoya Protocol

Arising from the recognition that biodiversity provides irreplaceable and priceless services to humankind, the climate, and the planet, the Nagoya Protocol (NP) was adopted on 29 October 2010 and entered into force in 2014 [22]. While a major part of genetic resources comes from developing countries, they have demanded the regulation of the utilisation of their genetic resources to ensure fair and equitable benefit sharing, which is the focus of the NP. The aim of the NP is to establish a framework that helps to access genetic resources for biotechnology research and development in return for a fair share of the benefits from their use. As of October 2021, the NP has been ratified by 132 UN member states and the EU [23]. In this respect, the EU has played a key role in the negotiations on the NP, as it is crucial for its Green Deal prospects and goals, as well as taking a leading role in the upcoming international negotiations on a new global framework to halt biodiversity loss. Since the USA did not participate in the negotiations as it is not a Party to the CBD, the EU represents approximately half of the utilisation of genetic resources to be regulated. Thus, the EU, together with Brazil, Norway, and several African countries, formed part of the core group in the final negotiations of the NP [24]. Due to the EU’s in-depth involvement, it has managed to achieve most of its policy objectives concerning the NP, particularly by adopting a Strategic Plan for Biodiversity 2011–2020 [24].

The NP is a supplementary agreement to the CBD to create greater legal certainty and transparency for both providers and users of genetic resources. The NP was created to: (1) ensure benefit sharing and (2) provide incentives to conserve genetic resources and their sustainable use, thus enhancing the biodiversity contribution to economic growth and human well-being [25]. The NP is important to the BB sector because it aims at creating legal certainty and transparency for both providers and users of genetic resources by: (1) establishing more predictable conditions for access to genetic resources and (2) helping to ensure benefit sharing when genetic resources leave the providing country (the donor).

The CBD and NP are international biodiversity-related legislation, and they recognise that biodiversity provides value to humankind and that the provider countries have sovereign rights over their TK and GR. In other words, the NP aims at providing access to genetic resources under an international system that fairly and equitably guarantees the benefit sharing of the GR of the provider country, both for the results of R&I and the benefits from the commercialisation of the GR after it leaves its borders [25].

The NP has 36 Articles to promote the use of genetic resources and the associated traditional knowledge; to strengthen the opportunities for the fair and equitable sharing of benefits from GR use; and that aim at creating incentives to conserve biological diversity and the sustainable use of its components, and to further enhance the contribution of biological diversity to sustainable development and human well-being, while safeguarding the rights of indigenous communities. Thus, the Contracting Parties are to: (1) create, implement, and enforce measures providing that GR utilised within their jurisdiction have been accessed by prior informed consent and that mutually agreed terms have been established, as required by another contracting party; (2) define cooperation regulations in cases of an alleged violation of the requirements of another contracting party; (3) encourage the drafting of contractual provisions on dispute resolution in mutually agreed terms; (4) ensure that an opportunity is available to seek recourse under their legal systems when disputes arise from mutually agreed terms; (5) take appropriate measures regarding access to justice; and (6) take measures to monitor the utilisation of genetic resources after they leave a country, including designating effective checkpoints at any stage of the value chain, i.e., research, development, innovation, pre-commercialisation, and/or commercialisation. These steps are covered by the following three legal agreements that must be drafted between the donor or provider and the user: (1) Prior Informed Consent (PIC), which is the “permission given by the Competent National Authority (CNA) of a country to an individual or institution seeking to obtain access to genetic resources, in line with an appropriate legal and institutional framework” [26]; (2) the Material Accession Agreement (MAA) or Mutually Agreed Terms (MAT), which is about the deposit and registration in a culture collection. This agreement states the conditions of access, use of the resources, and the benefits to be shared between both the donor and the user [26]. Finally, (3) the Material Transfer Agreement (MTA) is the agreement concerning the purchasing of biospecimen material from a culture collection (this is the recipient’s perspective) and providing said material (provider’s perspective) [27].

### Steps to Comply with the Nagoya Protocol and the Convention on Biological Diversity

In BB, the most common types of facilities that collect, process, store, and distribute biospecimens from other countries of origin (often referred to as the Provider or Donor) are Environmental Biospecimen Repositories (EBRs) and biomaterial repositories (referred to as the Depositors). The EBRs manage specimens from animals, other living organisms, and/or materials derived from biospecimen processing (i.e., DNA, proteins, enzymes, chemical entities extractions) (referred to as Genetic Resources) [28,29,30].

**Figure 1:**
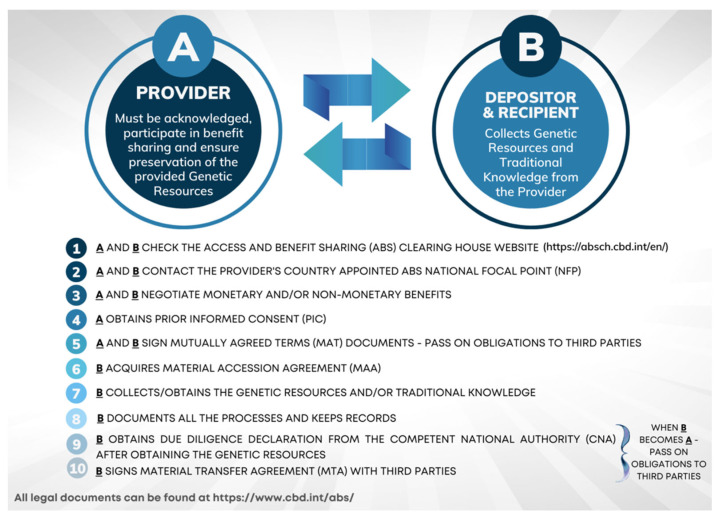
Steps to achieve legal, equal, and ethical sharing of genetic resources in Blue Biotechnology (based on “Necessary Legal Policy for Culture Collections” [31], https://absch.cbd.int/en/, accessed on 21 March 2022; https://www.cbd.int/abs/, accessed on 21 March 2022).

To meet the legal requirements set out by the NP, the CBD, and ABS frameworks among others, the Depositors, Providers, and Recipients (researchers, innovators, and other stakeholders, such as industry end-users) can use the following steps as presented in Figure 1, from the stage of sourcing Genetic Resources to their flow to the biotechnology field and industry [31]. In short, the Provider and Depositor sign agreements after negotiations to protect the former’s rights, but also to provide legitimacy to the latter. The signed agreements are the Mutually Agreed Terms (MAT) and the Material Accession Agreement (MAA). Additionally, the Depositor is obligated to contract a Material Transfer Agreement (MTA) with the Provider to gain permission to distribute to third parties the raw and/or processed biomaterials derived from the acquired Genetic Resources. The final users (Recipients) of said biomaterials can be various stakeholders, including the industry sector, that are also obligated to follow and respect ABS and RRI frameworks [31].

## 4. Overview of the Responsible Research and Innovation (RRI) Concept

As already mentioned, the European Biodiversity Framework focuses on co-responsibility and co-ownership, which is well-fitting with the Responsible Research and Innovation (RRI) concept. The EC has defined RRI as a science-policy framework that seeks to align innovations with societal needs and values. The overarching aim of RRI is to engage the citizens and other stakeholders in the research, development, and innovation processes to produce ethically acceptable, sustainable, and socially desirable R&I outcomes. Furthermore, RRI enables research organisations to become more aware of societal issues. Following the RRI principles may ensure a faster uptake of research outcomes and innovations. This is beneficial not only for the researchers and innovators, but also for the end-users and society in general. In a nutshell, RRI may increase citizen trust in R&I results and marketed products, as well as enable multi-way communication between science and society. Moreover, the benefits of embedding RRI in the R&I processes align with the latest communication of the EC “A new ERA for R&I” [32], specifying that Europe should increase its efforts in bringing R&I outcomes to the market. Hence, efforts need to be made to strengthen technology transfer, uptake of results, and industrial innovation, through knowledge transfer and public–private cooperation. Furthermore, the communication highlights that innovation ecosystems must be strengthened for knowledge circulation, valorisation, and public awareness as they are pivotal for the BB field and all sectors that depend on it. RRI may enable Innovation Ecosystems, which are business ecosystems that function like ecological ecosystems [33], where interconnected and interdependent innovative organisations are collaborating. Innovation Ecosystems form when universities, private and public research centres, small and medium-size enterprises (SMEs), and industry are collaborating to innovate. Moreover, Innovation Ecosystems depend on regional innovation strategies and regulatory agencies. Innovation Ecosystems form with conscious capacity building, co-creation, exploration, and exploitation activities [34], which links them very closely with the concept of RRI, where capacity building and co-creation are key elements.

Overall, RRI aims at supporting the institutional decisions concerning the goals of R&I by minimising ambiguity and uncertainty through anticipation, reflection, and alignment with end-users’ expectations and societal values. It means that research, development, and innovation become targeted and focused on resolving societal issues, such as food shortage, pollution, climate change, and diseases. In practical terms, RRI gives a regulatory frame for the researchers and the innovators with four dimensions to implement along the development of the BB value chain: anticipation, reflexivity, inclusion, and responsiveness [35]. Researchers and innovators may anticipate scenarios, technology assessments, risk analysis, life-cycle assessments, and socio-literary techniques. The goal of anticipation is to identify and appraise the possible and plausible impacts of R&I pathways and results. Researchers and innovators should have co-responsibility and co-ownership through co-creation with society and stakeholders on their work’s implications and socio-economic impacts. This can be achieved through multi-stakeholder partnerships, user-centred design, citizen and stakeholders focus groups, and similar participatory approaches. Reflexivity is about integrating innovation into society and the kind of effect this innovation/discovery can have in practice. Reflexivity requires multidisciplinary collaboration and the inclusion of social scientists in the R&I processes. Lastly, responsiveness is about policy and governance mechanisms for the practical implementation of responsible innovation [36]. This can be achieved through value-sensitive design, codes of conduct, transparency, regulation, and standards, and by relating R&I to societal challenges (e.g., health, food security). It is worth noting that most universities apply and understand RRI from a retrospective rather than a prospective point of view. In contrast to retrospective practices, prospective practices focus on value-laden dimensions of responsibility, asking what kind of products and services we want to see coming out of our science and innovation practices [37]. Therefore, more training on RRI awareness would be beneficial for young and experienced researchers in public/private/academic research centres.

### Implementation and Operationalisation of RRI by the European Commission (EC)

The EC has already embedded RRI in many R&I calls in the two funding programmes, FP7 [38] and Horizon 2020 [39]. RRI is continuing as a cross-cutting priority in the funding programme Horizon Europe (2021–2027) [40] under terms such as public engagement, societal engagement, innovative governance models, socially and environmentally responsible behaviour, and social innovation. The EC sees the RRI framework as the tool for engaging all societal actors (i.e., researchers, citizens, policy makers, policy implementers, industry, civil society organisations (CSOs), and non-governmental organisations (NGOs)) in the co-definition, co-design, co-creation, and co-construction of innovations that will help to sustainably resolve societal problems.

The EC has operationalised RRI through five dimensions: public engagement, open science, science education, gender equality and issues, and ethics. In RRI, governance is considered the overarching paradigm. In greater detail: (1) Public engagement [41] encompasses the involvement of the stakeholders early on in R&I processes to ensure that the results meet societal needs and expectations. (2) Open Science/Open Access [42] covers free access to publications and open science, meaning that the scientific results should be openly available, as they are financed by public money. Horizon Europe strengthened this concept [43] by establishing that, if not fully exploited one year (with exception) after completion of an R&I project, results should be made open to be utilised by other scientists [44]. (3) Science education is about informal and formal education. Both types are necessary. Informal education focuses on educating the public about the scientific field, creating awareness about the science and innovation processes. On the other hand, formal science education is necessary for future generations of scientists. (4) Gender equality eliminates gender bias in both the research process and research content. The gender issue is about considering any gender-related differences concerning the R&I results. (5) Ethics is about ethically performing research and development, respecting societal values, and keeping the highest standards of ethics and integrity for the performance and governance of R&I in the EU [45]. For ethics, the Vancouver Convention further entails compliance with the Helsinki Declaration, and independent committees must advise research projects on ethics. To this end, most R&I proposals focusing on biotechnology and requesting funding from the EC are undergoing an ethics evaluation. Ethics has been further strengthened in Horizon Europe with the DNSH principle, aligning with the European Green Deal objectives. According to the DNSH principle, R&I activities should carry activities that do not cause any significant harm to any of the six environmental objectives: climate change mitigation, climate change adaptation, sustainable use and protection of water and marine resources, transition to a circular economy, pollution prevention and control, and protection and restoration of biodiversity and ecosystems. This requirement is further specified in Article 17, on establishing a new framework, the EU Taxonomy Regulation, which aims to facilitate sustainable investments. Compliance with the DNSH principle needs to be assessed for activities carried out during an R&I project and the expected life-cycle impact of the innovation at a commercialisation stage. The DNSH principle is assessed during the project proposal evaluations and the execution of the project.

There are several ways of operationalising and implementing RRI. One methodology for RRI application is to follow the comprehensive RRI Roadmap [46], which embeds RRI with Systemic Innovation, Design Thinking, and Change Management approaches. The RRI Roadmap focuses on engaging stakeholders in an R&I project from its early stages and guiding them through a transition to early adoption of the innovation (product, service, or both). It provides a step-by-step methodology and tools. The RRI Roadmap is based on the lessons learned and best practices of 45 mobilisation and mutual learning workshops with more than 1000 stakeholders interested in Blue Economy and BB (2017–2018). Furthermore, on the RRI Tools platform, one finds several tools that can be applied for embracing RRI.

## 5. CA18238 Ocean4Biotech’s Survey Analysis

BB researchers, innovators, and other stakeholders are not limited by the boundaries of their own country nor the EU’s borders, as bioprospecting can take place all over the world. However, the three surveys conducted by CA18238 Ocean4Biotech’s Work Group 4 among its 130 members and their institutions in the EU member states and EU-associated countries, representing a total of 37 countries, showed that both young and experienced BB researchers, innovators, and other stakeholders seem to lack awareness of EU’s existing frameworks and regulations, including the NP and concepts such as RRI.

**Figure 2:**
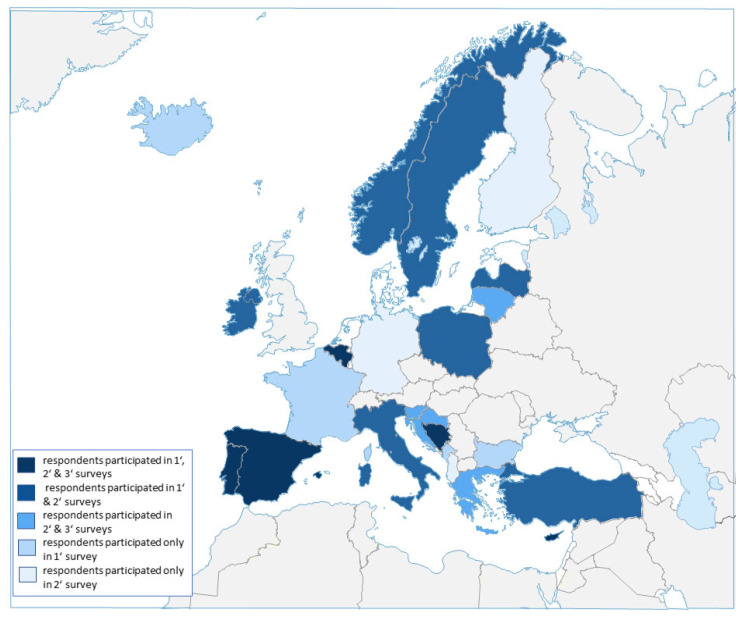
Countries that participated in one, two, or all three surveys: (1) RRI awareness and application; (2) NP awareness and knowledge; and (3) the extent of NP implementation in EU member states and associated countries.

Figure 2 highlights the countries in which the respondents are employed and whether they participated in one, two, or all three surveys.

During the activities and discussions conducted in the Ocean4Biotech meetings, it became evident that most of the researchers participating in the action were not aware and/or lacked knowledge regarding legislative tools and relevant guidelines. Thus, training and webinars were organised to raise awareness among the Ocean4Biotech BB community. In this context, to better understand the degree of lack of awareness of RRI and the NP, we conducted three surveys targeting the Ocean4Biotech participants and their institutions to investigate: (1) the knowledge and application of RRI; (2) the awareness and knowledge about the NP and; (3) the extent of NP implementation in EU member states and associated countries.

### 5.1. Survey 1: Awareness and Application of RRI in the Ocean4Biotech Network of Researchers

**Figure 3:**
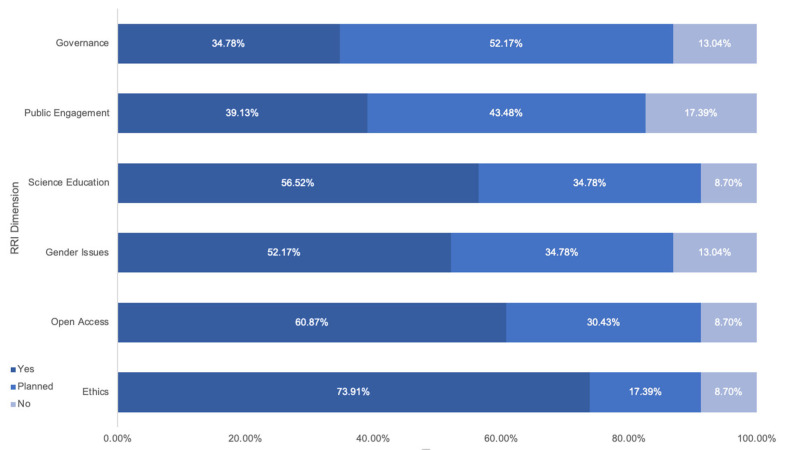
Percentage of Ocean4Biotech respondents who answered yes, planned to, or no to the question, “Are you already applying each of the Responsible Research and Innovation dimensions in your project/work?”.

After the webinar titled “Responsible Research and Innovation (RRI), and Intellectual Property Rights” that was conducted in June 2021, a survey was circulated among the 57 webinar participants to investigate how well they understood and applied RRI. Of the 57 participants, 23 responded, out of which 91.3% answered that they were aware of the RRI concept and principles. Looking deeper into how they apply RRI in their R&I processes, the majority focus on ethics, open access, gender issues, and science education, as illustrated in Figure 3. Specifically, 73.91% reported applying ethics in their work, and 60.87% are applying open access to their work. Only 52.17% of the respondents confirmed giving attention to gender issues during their research. Researchers were also asked if their institution had an operational Gender Equality Plan. Less than half of the participants, 47.8%, said their institution had such a plan, 39.1% said no, and 13.0% answered with a maybe.

Concerning the implementation of science education, 56.5% answered affirmatively. Concerning public engagement related to stakeholder involvement in the R&I processes for co-creation, only 39.13% answered yes, but 43.48% replied that they were planning to do so. For the overarching RRI dimension of governance, only 34.78% of the researchers responded that they applied it, 52.17% responded that they planned to do so, and 13.04% answered no. It is encouraging to see that the majority focus on ethics, followed by open access and gender issues. Perhaps an explanation for this is that ethics are already embedded to some degree in the curricula of universities, and research institutions focus on ethics as part of their organisational values. Concerning open access and gender issues, an explanation may be that they are both required in EU-funded projects and that proposals are measured against these three criteria: ethics, open access, and gender issues.

Asking the researchers about which RRI dimensions are necessary for research, the most-answered by order of importance were: Ethics (14), Science Education (12), Open Access (10), Governance (9), Gender Equality (6), and Public Engagement (4).

It is also worth noting that public engagement, which is about engaging the wider public for co-creation and innovation targeting societal problems, had the lowest ranking. It is interesting to note that public engagement, which was a requirement in Horizon 2020 and is also in Horizon Europe funding research calls, received the least focus from the BB community. When asked about which RRI dimensions are part of their institution’s policy, again Ethics scored the highest (16), followed by Science Education (13), Gender Equality (12), Governance (10), Public Engagement (9), and Open Access (7). What is worth perceiving is that the answers regarding the question of their own practice and the question about their institution’s policy were very different, which indicates that their opinion about their research does not necessarily match their institution’s policy. The survey indicates that researchers need more elucidation, awareness, and training on the RRI concept before they are able to embed it in their R&I processes.

### 5.2. Survey 2: Awareness and Knowledge about the Nagoya Protocol in the Ocean4Biotech Network of Researchers and Practitioners

To investigate the level of knowledge and application of the NP’s articles and provisions from key scientific institutions that delve into the BB and/or biodiscovery field, a survey consisting of 64 questions was handed out in electronic form to all the Ocean4Biotech COST Action members, where 42 out of the 130 members filled out the questionnaire, representing 19 EU member states and EU-associated countries (Figure 2). From all 42 participants, 27 represented the research institutions that employed them. Most of the questionnaire respondents were employed in EU member states (73.7%), and they were part of a university’s staff (71.4%), while the majority of the biospecimen/biomaterial collections handled were reported to be for taxonomic purposes (64.3%). The majority of the participants (71.4%) come from academic institutions (universities), and the other 28.6% of participants are either from public or private research institutions and private companies.

**Figure 4:**
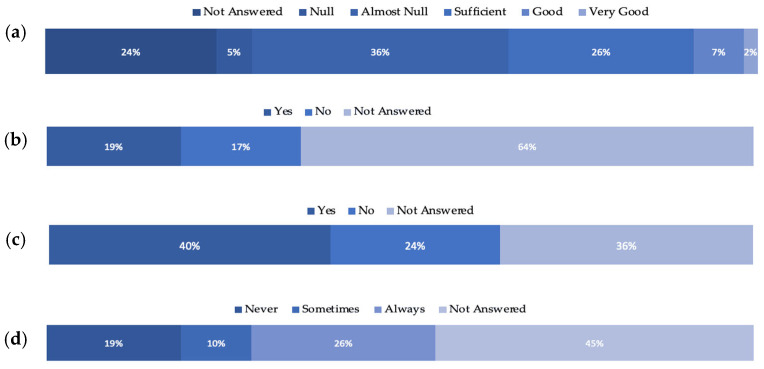
Percentage of Ocean4Biotech respondents for (**a**) awareness and knowledge of the NP; (**b**) using a Material Accession Agreement (MAA) when transferring samples between countries; (**c**) using Material Transfer Agreements (MTAs) for the exchange of samples; and (**d**) using MTAs for the supply of bio-samples/biospecimens.

The distributed questionnaire was sectioned in seven categories, namely: (1) General Information; (2) Sampling activities or working on samples coming from outside their country; (3) Regulations; (4) Access to the Samples; (5) Nagoya Protocol Knowledge; (6) Collection of Bio-samples/Biospecimens Information; and (7) Digital data. Twenty-four (24) questions from all categories were analysed, and the results are presented in Figure 4a–d. Only 9% of the participants described their knowledge and awareness on the Nagoya Protocol as “Very Good” and “Good”, where the majority (41%) stated that they have “Null” and “Almost Null” knowledge and awareness (Figure 4a). Most of the respondents opted not to answer (64%) when asked if they knew whether any of their collection specimens/strains originated from other countries and whether they were regulated under a Material Accession Agreement (MAA) (Figure 4b). Most of the respondents stated that they use a Material Transfer Agreement (MTA) for the exchange of any samples (40%) (Figure 4c), but only 26% for the supply of samples (Figure 4d).

Based on the survey’s results, there is a noticeable lack of knowledge about the NP, let alone its provisions. Almost in all categories of questions, the highest percentage was noticed in the “Not-Answered” field, which suggests that the participant either was unsure of what to apply at the institution/section where they work or did not want to share the information on the specific matter. These results highlighted the lack of knowledge about the NP’s implementation and its required enforcement in the BB community.

Regarding the use of MTAs, it was noticed that respondents do not apply them to the extent that the NP requires, especially when it comes to the supply of samples (Figure 4d), indicating a lack of implementation of the NP Article 5 “Fair and equitable benefit-sharing”. Additionally, the negotiations concerning participation in the benefits in case of commercial use of the biological material were also low among the respondents.

It may be concluded from the Ocean4Biotech survey that the overall awareness-raising actions and measures must be intensified regarding the importance of GR and TK associated with genetic resources, and the related access and benefit-sharing issues, as described in the NP Article 21. Since awareness actions and measures must be implemented at the national and subnational level of each Contracting Party of the NP, their inadequate application and enforcement may explain the results showing a low level of benefit-sharing awareness and understanding by the BB community and within their institutions, as observed in this survey.

### 5.3. Survey 3: The Extent of NP Implementation in EU Member States and Associated Countries

Building upon the collected responses to Survey 2, another investigation was conducted to gain a deeper insight into the NP implementation status in various EU member states and associated countries. The scope of Survey 3 was to investigate the structure and nature of the national set-ups supporting the implementation of the NP and to identify the barriers and enablers within the implementation process. A total of 11 researchers from nine European countries responded and completed the Survey 3 questionnaire regarding the implementation status of the NP. Unfortunately, none of the participants who claimed to have very good or good knowledge about the NP responded to this survey. Similarly, none of those who claimed to have no knowledge (Null) of the NP in Survey 2 responded to Survey 3. Five participants that claimed to have a sufficient knowledge level about the NP in Survey 2 completed Survey 3, as well as six participants who answered with “Almost null” in Survey 2.

A variety of scenarios were observed that indicate the disparity of stages in which EU member states and associated countries have implemented the NP. According to the claims, 54.55% of participants work only with local samples and have never attempted to contact their NP National Focal Point (NFP) or tried to apply for a permit under the NP. A lack of time to become familiar with the NP was reported by one respondent. Still, it was unclear whether the respondent’s work involved samples shared outside of their resident country. Other respondents said that “they shared the samples with foreign colleagues but did not comply with the NP requirements because the procedure was unclear” or “they did not know that the NFP office existed or that any information was nationally available”. Two respondents shared that they attended several informative sessions about the NP organised by the country’s NFP and perceived information about the permit application as clear and straightforward. However, they received no response to their application, and no permits were issued even months after the application submission. Although some EU countries are not a party to the NP, one local research team asked for written permission to sample strictly protected species. According to the respondent, the responsible governmental body was interested only in the sampling process, not whether the samples would be shared abroad. Finally, two other participants stated that there was no readily available information on the NP from the Competent National Authority offices nor from the faculties and the administration participating in the protocol implementation.

As part of Survey 3, an investigation was conducted about what publicly available information exists in the EU member states and associated countries regarding the implementation of the NP. The results of this investigation are summarised in Table S2. The survey showed that only 3 (12%) out of 25 investigated NP-signee countries have ABS procedures and application forms available online. However, more than half (14 countries) have national websites with information about the NP, due diligence, a checklist for BB researchers, innovators, and other stakeholders, and legal information. We could not find any available information regarding the NP implementation protocols for several countries, which was in line with the participants’ statements from these respective countries.

The results of Surveys 2 and 3 regarding the application and the implementation of the NP were in agreement. A noticeable lack of knowledge about the NP and its execution conditions was recorded among the targeted group. As expected, this situation was not reserved only for Inclusiveness Target Countries (ITC) but also for research-intensive countries. Improving communication and dissemination from the NFPs about the conditions required for national NP compliance and how to increase awareness and application of the NP within the scientific community are crucially needed. A lack of feedback from NFPs, even when contacted, is hampering the progress of R&I projects. Hence, an NFP’s failure to issue a permit (or give guidelines about correctly applying for authorisations) for an extended time is not compatible with any R&I project timeline. This situation may be explained due to the fact that most appointed NFPs are administrative staff members and not specialists, and most likely do not have enough resources to effectively run the application process.

**Figure 5:**
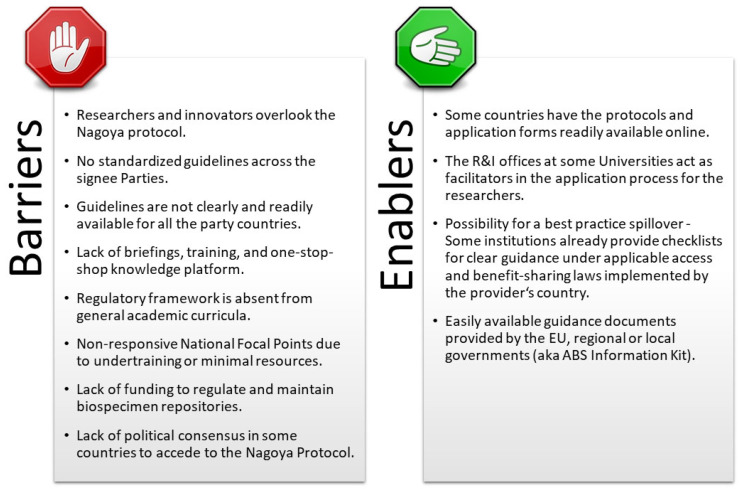
Barriers and enablers in the implementation of the NP identified from the conducted survey.

Barriers and enablers in implementing the NP, as recognized from the responses to Survey 3, are highlighted in Figure 5.

## 6. Recommendations to the Blue Biotechnology Community for the Application of the NP and RRI Framework

Herein, we provide recommendations based on the gaps identified by the three surveys in the Ocean4Biotech community’s awareness, knowledge, and application of RRI and regulatory frameworks. The gaps can be divided into five main categories: awareness, understanding, education, implementation, and enforcement. The suggested recommendations and calls for future actions targeting the main stakeholders in the BB community are stated in the following text.

### 6.1. Recommendations to EU Policy Makers

It is proposed that a pan-European Blue Biotechnology regulatory knowledge hub be created with the support of the EC. This knowledge hub may be the beginning of an online Blue Biotechnology Innovation Ecosystem that would provide one-stop-shop information for researchers and innovators. This knowledge hub may include international, EU, and national regulations, guidelines, tools, facilities, products, funding information, projects, results, innovations, databases, sample banks, etc. Thus, a pan-European Blue Biotechnology knowledge hub may further enable cooperation among European regions to increase BB as a Key Enabling Technology sector to strengthen Europe’s competitive position and job creation.

Linking EU policies and directives to the NP and its sister protocols and conventions should be considered, so that they become evident and known.

The DGs dealing with BB may consider creating a community of practice where the EU regulations and the NP are discussed and knowledge exchange takes place among the community members. Creating a community of practice is not new to the EC, where several exist. Two excellent examples are the community concerning security matters, CERIS (Community of European Research and Innovation for Security), which dedicates knowledge exchange among others on the Sendai Framework on Disaster Risk Reduction, and the Water Europe community, where water frameworks are also discussed. Such a BB community of practice will enrich the R&I initiatives and increase the awareness and exchange of knowledge regarding BB regulations and RRI.

The collaboration among the NFPs of the EU member states and associated countries should be increased, with annual briefing sessions for researchers and innovators concerning EU and international legal frameworks such as the NP.

### 6.2. Recommendations to Governments or Competent National Authority

National governments, as the first and foremost authorities responsible for implementing the NP, should also actively participate in improving the level of awareness, for example, by easing administrative procedures and overall reinforcing the Nagoya Protocol.

Governments should conduct a self-assessment to evaluate their efficiency in implementing the NP and identify and rectify gaps. Based on this self-evaluation, governments need to invest in improving awareness and make information easily accessible via actions of dissemination, education, and training with community involvement, and through the means of, for example, webinars, workshops, conferences, or use of media, among others.

National websites must be enriched with readily available information and application forms. NFPs must step up offering supporting activities and awareness and training initiatives. Hence, the NFPs’ own training must be improved. NFPs must be evaluated with respect to their responsiveness, maximum delay of response, and/or support towards researchers and innovators.

Administrative processes should be transparent and easily accessible, with a fast turnaround. Funding ought to be envisioned to enable the proper registration and maintenance of biospecimen repositories/collections.

Finally, effective enforcement can be achieved via audits at all levels, as it is currently practised in some sectors, such as the pharmaceutical industry. Standardisation of biotechnological methods, including ethics certification and regulatory education, are also suggested.

### 6.3. Recommendations to Researchers and Innovators

Researchers and innovators are at the heart of accessing genetic resources and are, therefore, the main responsible body for implementing RRI and regulatory frameworks such as the NP.

Researchers should check the Access and Benefit-Sharing (ABS) Clearing-House website and/or contact the country’s appointed ABS National Focal Point. In situations where there is little or no external help and support, the researcher’s own ethics for compliance with the policies and regulations should prevail. Researchers have the responsibility to self-educate on the topic.

### 6.4. Recommendations to Academia

Academia needs to introduce the RRI framework and tools in their curricula and R&I processes, as well as comply with EU and national policies and regulations such as the NP. The introduction of metrics for evaluating their impact on sustainable R&I activities, followed by periodical environmental and laboratory audits, should be considered. Researchers and other individuals who access genetic resources receive their education in academia. Hence, academic courses and certifications present an excellent opportunity to educate young and established researchers about resource sharing and the NP. Therefore, courses and certifications concerning regulatory frameworks, RRI, and ethics should also be embedded in academic studies, coupled with sustainability and biodiversity courses. These courses may be open not only to the university’s students but to other academic institutions and individuals from industry; thus, they can become an extra source of income for the university.

Several academic institutions have teams to provide assistance and guidance to their researchers. However, a great number of institutions need to address the lack of awareness and knowledge highlighted in this perspective paper.

### 6.5. Recommendations to Industry

Private and public research centres, large companies, and small and medium-size enterprises (SMEs) need to realise the importance of compliance to regulations and the benefits of following the RRI approach as part of their institutional governance and societal responsibility. This awareness must be extended to their shareholders so that they may also understand their green and blue responsibilities towards society and the environment at large. One way to achieve it is by embedding it in the organisational ethos and values, as well as by extending their financial reporting with sustainability actions and considerations.

Industry can embed RRI and the EU regulations, including the NP, in their research standard operating procedures and provide training to their employees as part of their quality assurance procedures. For example, by understanding that the RRI dimensions of public engagement and gender issues enable market segmentation and deeper knowledge of needs and expectations, companies gain without expensive outsourced market research studies. Thus, companies may see the application of RRI and regulatory frameworks as investments for securing the future of their products and services. Furthermore, many investment organisations, such as pension companies, are focusing more and more on green and blue opportunities that are responsible and sustainable. In addition, the institutions should allocate compulsory resources to ensure that employees apply the RRI approach and follow EU regulations.

### 6.6. Recommendations to Incubators and Smart Regions

Incubators may also support young start-ups in the early understanding of necessary regulations and good RRI practices. This is possible through training and vouchers to enable start-ups and SMEs in this regard.

Smart regions can promote and enable increasing awareness and application of RRI and regulatory frameworks, including the NP, with training and the application through their activities as, for example, through their living labs.

### 6.7. Recommendations to Funding Bodies

Funding bodies may further accelerate regulatory compliance, as well as the application of RRI in R&I initiatives, by including them as conditions in calls for funding of R&I projects at the EU and national levels. Their evaluation will provide sufficient feedback to researchers, practitioners, and companies regarding their importance, who will then start focusing on their application if they wish to receive funds for their R&I actions.

### 6.8. Recommendations to Scientific Journals

Scientific journals may further accelerate regulatory compliance, as well as the application of RRI in R&I initiatives, by including them as conditions for publication acceptance. Their evaluation will provide sufficient feedback to researchers, practitioners, and companies regarding their importance, who will then start focusing on their application if they wish to publish their R&I outcomes.

### 6.9. Recommendations to COST CA18238 Ocean4Biotech Action

Members of the CA18238 Ocean4Biotech recognised that they have a role in increasing awareness about the RRI, EU regulations, and the NP. For the duration of the CA18238 Ocean4Biotech action, its members will continue, therefore, to conduct workshops, training, and dissemination via publications, policy briefs, talks, and other supportive materials (e.g., cartoons, videos, presentations, checklists). 

## 7. Materials and Methods

The three surveys were conducted in 2021: (1) knowledge and application of RRI in the Ocean4Biotech network of researchers; (2) awareness and knowledge towards the Nagoya Protocol; and (3) the extent of the Nagoya Protocol implementation in EU member states and associated countries.

Survey 1 about “Knowledge and Application of Responsible R&I in the Ocean4Biotech Network of Researchers” consisted of eight questions and was distributed via Google Forms to all 57 webinar participants regarding “Responsible R&I (RRI), and Intellectual Property Rights” held in June 2021.

Survey 2 about “Awareness and Knowledge towards the Nagoya Protocol” consisted of 64 questions and was distributed via Google Forms to all the Ocean4Biotech COST Action partner institutions and members. Out of the 130 COST members, 42 filled out the questionnaire, representing 19 EU member states and EU associated countries. The distributed questionnaire was sectioned into seven categories based on the questions’ theme: (1) General Information, (2) Sampling activities or working on samples coming from outside their country, (3) Regulations, (4) Access to the samples, (5) Nagoya Protocol Knowledge, (6) Collection of bio-samples/biospecimens information, and (7) Digital data.

Survey 3 about “The extent of the Nagoya Protocol implementation in EU member states and associated countries” was launched in October 2021. Electronic questionnaires were emailed to 28 Ocean4Biotech action members (representing 15 European countries) who answered the previous surveys’ question, “What is your awareness of the Nagoya Protocol?”. Participants were structured into two groups, depending on how they assessed their awareness level regarding the NP in the previous survey: Very Good, Good, or Sufficient (Group 1), and Almost Null or Null (Group 2). Both groups were asked whether they were willing to share a concrete example of the NP application in their research and their experience interacting with the National Focal Point (NFP) or Competent National Authority (CNA). The participants were asked to identify any barriers/enablers that facilitated/hampered the application of the NP for them (or their colleagues). Additionally, the second group was asked to elaborate the rationale to their answer regarding their awareness level (Almost Null or Null). Finally, we conducted an overview of national records regarding the implementation of the NP from the ABSCH (The Access and Benefit-Sharing Clearing-House) website to cross-check the data gathered from the respondents with the information available online. The availability of information regarding the appointed NFPs, openly accessible ABS procedures and application forms, and the existence of national websites or databases with further details regarding the implementation on a national level were assessed.

Through the analyses of these surveys, several gaps have been identified concerning ABS and NP awareness, which led to a set of recommendations presented in this perspective paper.

## 8. Conclusions

Six main gaps for legal and ethical R&I in Blue Biotechnology have been identified: awareness, understanding, education, implementation, enforcement, and auditing. It has become evident in the three surveys conducted by the Ocean4Biotech action that RRI and BB regulatory frameworks, such as the NP, are not fully understood and applied by BB researchers, as they see them more as extra work and disablers. To reinforce the awareness of the marine biotechnology community on the importance of implementing the NP, BB-related policies, and RRI frameworks, recommendations are provided per major stakeholder group, and the main recommendations to mitigate these awareness and compliance gaps are summarized below:

1. The European Commission should create a Blue Biotechnology community of practice accompanied by a knowledge hub for regulations and good practices;
2. Governments should improve awareness, ease of administrative, bureaucratic procedures, and overall enforcement of the Nagoya Protocol;
3. Academia and Industry must comply with EU policies and regulations by allocating human resources to help researchers and employees to implement the RRI approach in their research and follow EU regulations, including the Nagoya Protocol;
4. RRI and Ethics should be embedded early in academic courses;
5. Researchers and Industry should apply RRI at all times and comply with EU policies and regulations, including the Nagoya Protocol;
6. Compliance to regulations, including the Nagoya Protocol, may be part of investors’ due diligence practices.

## Supplementary Materials

The following supporting information can be downloaded at: https://www.mdpi.com/article/10.3390/md20050290/s1, Table S1: Overview of the regulatory guidelines to be considered during the BB value chain, from organism identification to natural product uptake; Table S2: Overview of the information on the appointed NFPs, online available ABS procedures and application forms, and national websites or databases with information regarding the NP implementation on a national level for EU members and associated countries.
