# Supplementary material for: Responsible Research and Innovation Framework, the Nagoya Protocol and Other European Blue Biotechnology Strategies and Regulations: Gaps Analysis and Recommendations for Increased Knowledge in the Marine Biotechnology Community
Source: Mar Drugs. 2022 Apr 26;20(5):290. doi: 10.3390/md20050290 (PMC9144305; doi:10.3390/md20050290)
Supplement: Supplementary file 1 [file marinedrugs-20-00290-s001.zip › marinedrugs-1668400-supplementary.pdf]

marine drugs

MDPI

Supplementary Materials

Why Every Marine Biotechnology Scientist Must consider the Responsible Research and Innovation, the Nagoya protocol and other European Blue Biotechnology strategies and regulations

Xenia Theodotou Schneider [1,*], Belma Kalamujić Stroil [2], Christiana Tourapi [3], Céline Rebours [4], Susana P. Gaudêncio [5,6], Lucie Novoveska [7] and Marlen I. Vasquez [3,8]

[1] XPRO Consulting Limited, Strovolos 2021, Cyprus
[2] Institute for Genetic Engineering and Biotechnology, University of Sarajevo, 71000 Sarajevo, Bosnia and Herzegovina; belma.kalamujic@ingeb.unsa.ba
[3] Department of Chemical Engineering, Cyprus University of Technology, Limassol 3036, Cyprus; ctourapi@yahoo.com (C.T.); marlen.vasquez@cut.ac.cy (M.I.V.)
[4] Møreforsking AS, 6021 Ålesund, Norway; celine.rebours@moreforsking.no
[5] Associate Laboratory i4HB, Institute for Health and Bioeconomy, NOVA School of Science and Technology, NOVA University Lisbon, 2819-516 Caparica, Portugal; s.gaudencio@fct.unl.pt
[6] UCIBIO—Applied Molecular Biosciences Unit, NOVA School of Science and Technology, NOVA University of Lisbon, 2819-516 Caparica, Portugal
[7] Ocean4Biotech, Edinburgh EH12 5AD, UK; lucie.novoveska@gmail.com
[8] European University of Technology, Limassol 3036, Cyprus
* Correspondence: xenia-schneider@xpro-consulting.com; Tel.: +33-6-69776917

Table S1. An overview of the regulatory guidelines to be considered during the BB value chain, from organism identification to natural product uptake.

Marine Biotechnology Value Chain Steps	European Union Directives, Policies and Guidelines	International Conventions, Protocols, Guidelines and Standards
**1. Organisms / Microorganisms Sourcing**<br>• *Ex situ*<br>• Biological Resource Centres<br>• *In situ*<br>• Harvesting from the marine environment	EC Regulations in force:<br>• Regulation (EC) No 1946/2003 of the European Parliament and of the Council of 15 July 2003 on transboundary movements of genetically modified organisms<br>• Marine Strategy Framework Directive<br>• Marine Protected Areas<br>• Regulation (EU) 2015/1866 of 13 October 2015 laying down detailed rules for the implementation of Regulation (EU) No 511/2014 of the European Parliament and of the Council as regards the register of collections, monitoring user compliance and best practices<br>• Sea-Basin Strategies (including the outermost regions in the Atlantic Ocean (including Macaronesia and Caribbean-Amazonia) and of its corresponding action plan 73)<br>• European Aquaculture Technology Platform for the improved management of the biological lifecycle<br>• Council Directive 98/58/EC of 20 July 1998 Concerning the Protection of Animals Kept for Farming Purposes. Off. J. L 1998, 221, 23–27 (https://eur-lex.europa.eu/eli/dir/1998/58/2019-12-14).<br>• Council Regulation (EC) No 1/2005 of 22 December 2004 on the Protection of Animals during Transport and	• Convention on Biological Diversity<br>• Nagoya Protocol<br>• Cartagena Protocol on Biosafety<br>• Bonn Guidelines (Bonn Guidelines on access to genetic resources and fair and equitable sharing of the benefits arising out of their utilization (cbd.int))<br>• Internationally recognized certificate of compliance: Prior Informed Consent (PIC), contact National Focal Points (NFPs) (https://absch.cbd.int/countries) and have documented permission from Competent National Authorities (CNAs)<br>• Mutually Agreed Terms (MAT), including benefit-sharing arrangements, between provider and user (which depends on the institutional guidelines).<br>• Material transfer agreement (for bioresource exchanging), which depends on the institutional guidelines.<br>• Antarctic treaty (att005_e.pdf ; The Antarctic Treaty | Antarctic Treaty (ats.aq))<br>• Svalbard treaty (International Agreements - Arctic Portal)<br>• Agreement on the Conservation of Cetaceans in the Black Sea Mediterranean Sea and Contiguous Atlantic Area

	Related Operations and Amending Directives 64/432/EEC and 93/119/EC and Regulation (EC) No 1255/97. Off. J. Eur. Union 2005, 1, 1–44 (https://eur-lex.europa.eu/eli/reg/2005/1/2019-12-14)https://eur-lex.europa.eu/eli/reg/2005/1/2019-12-14.<br>• Council Regulation (EC) No 1099/2009 of 24 September 2009 on the Protection of Animals at the Time of Killing (Text with EEA Relevance). Off. J. Eur. Union 2009, 303, 1–30 (https://eur-lex.europa.eu/legal-content/EN/ALL/?uri=CELEX%3A32009R1099).<br>• The Ecosystem Approach to the management of human activities that may affect the Mediterranean marine and coastal environment (in the framework of the Barcelona Convention)	
**2. Cultivation and Biomass Production**<br>• Use of Best Practices Protocols<br>• Biomass generated (production data, export, import)<br>• Libraries / Biobanks<br>• Specimens/Strains cataloguing<br>• Specimens/Strains preservation<br>• Specimen/Strain selection<br>• DNA for identification<br>• Selection of broodstock of Bioactive microorganisms (Genome Sequencing, gene expression etc.)	• 2000/608/EC: Commission Decision of 27 September 2000 concerning the guidance notes for risk assessment outlined in Annex III of Directive 90/219/EEC on the contained use of genetically modified micro-organisms (notified under document number C(2000) 2736<br>• Directive 2009/41/EC of the European Parliament and of the Council of 6 May 2009 on the contained use of genetically modified micro-organisms (Recast)<br>• Universal Declaration of Human Rights | United Nations and its international Human right laws.<br>• GLP (Good Laboratory Practice (europa.eu)) which include two EU directives : EUR-Lex - 32004L0009 - EN -	• Nagoya Protocol<br>• Internationally recognized certificate of compliance:<br>• Prior Informed Consent (PIC), contact National Focal Points (NFPs) and have documented permission from Competent National Authorities (CNAs)<br>• Mutually Agreed Terms (MAT), including benefit-sharing arrangements, between provider and user.<br>• Material transfer agreement (for bioresource exchanging), between provider and user.<br>• Mandatory Deposit DNA sequences: International Nucleotide Sequence Database Collaboration, which comprises the DNA DataBank of Japan (DDBJ), the European Nucleotide Archive (ENA), and GenBank at NCBI.

• Limited standing biomass per site and/or limited sea or land area for such production<br>• Lost/waste during production<br>• Processing of the product<br>• Documentation of the product quality and if used as food or feed ingredient, feed and food safety should apply<br>• Environmental impact of production<br>• Import of the material for this production<br>• Work conditions and worker security<br>• Biohazards (chemicals used for cleaning or to control diseases)	EUR-Lex (europa.eu) and EUR-Lex - 32004L0010 - EN - EUR-Lex (europa.eu)<br>• EU regulation on personal protective equipment 32016R0425 - EN - EUR-Lex	• Mandatory Deposit genome sequences: Integrated Microbial Genomes & Microbiomes (https://img.jgi.doe.gov/)<br>• ISO Laboratory Standards<br>• ISO Biotechnology — Ancillary materials present during the production of cellular therapeutic products:<br>o ISO/TS 20399-1:2018<br>o ISO/TS 20399-2:2018<br>o ISO/TS 20399-3:2018 ISO/CD 20399 Biotechnology — Ancillary materials present during the production of cellular therapeutic products and gene therapy products (will replace the ISO/TS 20399-3:2018)<br>• Conformity assessment procedures for protective equipment<br>• WHO Pandemic Influenza Preparedness Framework<br>• Biosafety in Microbiological and Biomedical Laboratories (BMBL) 6th Edition
**3. Discovery of organism / microorganism potential**<br>• Bioactivity screening<br>• Specimen/ Strain selection<br>• Right of ownership<br>• Use of Best Practices Protocols	• Directive 2009/41/EC of the European Parliament and of the Council of 6 May 2009 on the contained use of genetically modified microorganisms (Recast)<br>• IP Protection for genetically modified organisms / microorganisms, use of organisms / microorganisms for an innovative utility; process using organisms / microorganisms, screening / diagnostic assays<br>• European standards | Internal Market, Industry, Entrepreneurship and SEs	ISO Laboratory Standards<br>• Internationally recognized certificate of compliance:<br>• Prior Informed Consent (PIC), contact National Focal Points (NFPs) and have documented permission from Competent National Authorities (CNAs)<br>• Mutually Agreed Terms (MAT), including benefit-sharing arrangements, between provider and user.<br>• Material transfer agreement (for bioresource exchanging)

**4. Natural Product Discovery and IP Protection**<br>• Dereplication / Prioritisation of Extracts<br>• Extraction and Isolation of Natural Products<br>• Natural Product Structure Elucidation and Determination of Absolute l Configuration<br>• Pure Natural Product Bioactivity Screening<br>• Use of Best Practices Protocols<br>• Sustainable Natural Products scale-up production (synthesis and/or fermentation)<br>• Legal clarity for use<br>• Proof of compliance with regulations	• Chemicals legislation (europa.eu)<br>• Directive 98/44/EC on the legal protection of biotechnological inventions<br>• IP protection for innovative use of Natural Products[i]<br>• European Patent Agency and National Intellectual Property Agencies<br>• Sustainable Blue Economy Finance Principles (EC, EIB, WWF, ERI)	ISO Laboratory Standards<br>Sustainable Blue Economy Finance Principles (UNEP FI)<br>UN Environment's Principles for Sustainable Insurance Initiative (PSI) and the World Bank<br>Material transfer agreement
**5. Uptake for Research and Development in one of the following sectors:**<br>• Pharmaceutical<br>• Nutraceutical<br>• Cosmetics<br>• Food<br>• Chemical Industry<br>• Energy<br>• Environment<br>• Textile<br>• Naval	• Regulations based on each sector	• Regulations based on each sector

Table S2. An overview of the information on the appointed NFPs, online available ABS procedures and application forms, and national websites or databases with information regarding the implementation on a national level for EU members and associated countries. Abbreviations: NA – Not applicable.

No.	Country	NCP URL	Website with ABS Procedure	Website with the Forms	Application Fees Exist	National Websites or Database
**1.**	Austria	https://www.cbd.int/kb/record/focalPoint/264	NA	NA	NA	http://www.biodiv-abs.at/; http://www.biologischevielfalt.at
**2.**	Belgium	http://www.cbd.int/kb/record/focalPoint/6528	NA	NA	NA	http://nagoya.vlir.be/en/home-tool/; https://www.wallonie.be/fr/demarches/sinformer-sur-le-protocole-de-nagoya-et-ses-obligations; http://www.biodiv.be/convention/nagoya-protocol-access-and-benefit-sharing
**3.**	Bulgaria	http://www.cbd.int/kb/record/focalPoint/7394	NA	NA	NA	NA
**4.**	Croatia	http://www.cbd.int/kb/record/focalPoint/7642	NA	NA	NA	NA
**5.**	Cyprus	http://www.cbd.int/kb/record/focalPoint/5934	NA	NA	NA	NA
**6.**	Czech Republic	http://www.cbd.int/kb/record/focalPoint/6895	NA	NA	NA	https://www.mzp.cz/cz/nagojsky_protokol
**7.**	Denmark	http://www.cbd.int/kb/record/focalPoint/2410	NA	NA	NA	https://eng.mst.dk/nature-water/nature/biodiversity-the-building-block-of-life/the-nagoya-protocol-on-access-and-benefit-sharing/; https://mst.dk/natur-vand/natur/biodiversitet/hvordan-bevarer-vi-biodiversiteten/globalt-2020-maal/abs-protokol/
**8.**	Estonia	http://www.cbd.int/kb/record/focalPoint/7257	NA	NA	NA	NA
**9.**	Finland	http://www.cbd.int/kb/record/focalPoint/6090	NA	NA	NA	http://www.biodiversity.fi/geneticresources/home
**10.**	France	http://www.cbd.int/kb/record/focalPoint/5585	https://absch.cbd.int/database/PRO/ABSCH-PRO-FR-249352; https://absch.cbd.int/database/PRO/ABSCH-PRO-FR-249353; https://absch.cbd.int/database/PRO/ABSCH-PRO-FR-249354	https://www.formulaires.service-public.fr/gf/cerfa_15784.do; https://www.formulaires.service-public.fr/gf/cerfa_15785.do; https://www.formulaires.service-public.fr/gf/cerfa_15786.do	No	• https://webgate.ec.eu-ropa.eu/declare/web/domain; • http://www.ensei-gnementsup-recher-che.gouv.fr/cid127438/les-plates-formes-d-enregistrement-pour-l-utili-sation-de-ressources-genetiques-et-de-connaissances-traditionnelles-as-sociees.html; • https://www.ecologique-sol-idaire.gouv.fr/acces-et-partage-des-avantages-decoulant-lutilisation-des-ressources-genetiques-et-des-connais-sances
**11.**	Germany	http://www.cbd.int/kb/record/focalPoint/7089	NA	NA	NA	http://abs.bfn.de
**12.**	Greece	http://www.cbd.int/kb/record/focalPoint/7486	NA	NA	NA	NA
**13.**	Hungary	http://www.cbd.int/kb/record/focalPoint/2662	NA	NA	NA	http://www.biodiv.hu
**14.**	Ireland	http://www.cbd.int/kb/record/focalPoint/6384	NA	NA	NA	NA
**15.**	Italy	http://www.cbd.int/kb/record/focalPoint/7549	NA	NA	NA	NA
**16.**	Latvia	http://www.cbd.int/kb/record/focalPoint/5785	NA	NA	NA	NA
**17.**	Lithuania	http://www.cbd.int/kb/record/focalPoint/4167	NA	NA	NA	NA
**18.**	Luxembourg	http://www.cbd.int/kb/record/focalPoint/7090	NA	NA	NA	NA

**19.**	Malta	http://www.cbd.int/kb/record/focalPoint/3766	https://absch.cbd.int/database/PRO/ABSCH-PRO-MT-248287	https://absch.cbd.int/api/v2013/documents/076627E8-00C9-4696-2DDD-4FA6077AF1F0/attachments/GRU-005%20PIC%20Application%20-%20AC.docx; https://absch.cbd.int/api/v2013/documents/076627E8-00C9-4696-2DDD-4FA6077AF1F0/attachments/GRU-003%20PIC%20Application%20-%20RD.docx	yes	https://agrifish.gov.mt/en/phd/
**20.**	Netherlands	http://www.cbd.int/kb/record/focalPoint/6745	NA	NA	NA	http://www.absfocalpoint.nl
**21.**	Poland	http://www.cbd.int/kb/record/focalPoint/2423	NA	NA	NA	NA
**22.**	Portugal	http://www.cbd.int/kb/record/focalPoint/4871	NA	NA	NA	http://www.icnf.pt/portal/pn/biodiversidade/ei/cbd/prot-nagoia
**23.**	Romania	http://www.cbd.int/kb/record/focalPoint/5765	NA	NA	NA	NA
**24.**	Slovakia	http://www.cbd.int/kb/record/focalPoint/6680	NA	NA	NA	NA
**25.**	Slovenia	http://www.cbd.int/kb/record/focalPoint/5880	NA	NA	NA	NA
**26.**	Spain	http://www.cbd.int/kb/record/focalPoint/5963	NA	NA	NA	https://www.miteco.gob.es/en/biodiversidad/temas/recursos-geneticos/protocolo-de-nagoya/FAQ.aspx
**27.**	Sweden	http://www.cbd.int/kb/record/focalPoint/4187	NA	NA	NA	http://www.swedishepa.se/Guidance/Guidance/Species-Protection/Genetic-resourses/
**28.**	Iceland	NA	NA	NA	NA	NA
**29.**	Liechtenstein	NA	NA	NA	NA	NA
**30.**	Norway	http://www.cbd.int/kb/record/focalPoint/7516	NA	NA	no	Nagoya-protokollen I Forskningsetikk
**31.**	Switzerland	http://www.cbd.int/kb/record/focalPoint/6873	https://absch.cbd.int/database/PRO/ABSCH-PRO-CH-252013; https://absch.cbd.int/database/PRO/ABSCH-PRO-CH-252012	https://www.bafu.admin.ch/dam/bafu/fr/dokumente/biotechnologie/formular/formular_fuer_diemeldungeineszugangszugenetischenressourceniminl.docx.download.docx/formulaire_pour_lanot	no	https://www.bafu.admin.ch/bafu/en/home/topics/biotechnology/info-specialists/nagoya-protocol.html
**32.**	Albania	http://www.cbd.int/kb/record/focalPoint/5135	NA	NA	NA	NA
**33.**	Montenegro	http://www.cbd.int/kb/record/focalPoint/6312	NA	NA	NA	NA
**34.**	North Macedonia	http://www.cbd.int/kb/record/focalPoint/5663	NA	NA	NA	NA
**35.**	Serbia	http://www.cbd.int/kb/record/focalPoint/5019	NA	NA	NA	NA
**36.**	Turkey	http://www.cbd.int/kb/record/focalPoint/7445	NA	NA	NA	NA
**37.**	Bosnia and Herzegovina	http://www.cbd.int/kb/record/focalPoint/3523	NA	NA	NA	NA
